# Identification and characterization of new isoforms of human fas apoptotic inhibitory molecule (FAIM)

**DOI:** 10.1371/journal.pone.0185327

**Published:** 2017-10-05

**Authors:** Elena Coccia, Isabel Calleja-Yagüe, Laura Planells-Ferrer, Blanca Sanuy, Belen Sanz, Joaquin López-Soriano, Rana S. Moubarak, Francina Munell, Bruna Barneda-Zahonero, Joan X. Comella, M. Jose Pérez-García

**Affiliations:** 1 Cell Signaling and Apoptosis Group, Vall d´Hebron Institute of Research (VHIR), Barcelona, Spain; 2 Centro de Investigación Biomédica en Red sobre Enfermedades Neurodegenerativas (CIBERNED), ISCIII, Madrid, Spain; 3 Institut de Neurociències, Departament de Bioquímica i Biologia Molecular, Facultat de Medicina, Universitat Autònoma de Barcelona, Bellaterra, Spain; 4 Pediatric Neurology Group, Vall d´Hebron Institute of Research (VHIR), Barcelona, Spain; Universidad de Castilla-La Mancha, SPAIN

## Abstract

*Fas Apoptosis Inhibitory Molecule* (*FAIM*) is an evolutionarily highly conserved death receptor antagonist, widely expressed and known to participate in physiological and pathological processes. Two *FAIM transcript* variants have been characterized to date, namely FAIM short (FAIM-S) and FAIM long (FAIM-L). FAIM-S is ubiquitously expressed and serves as an anti-apoptotic protein in the immune system. Furthermore, in neurons, this isoform promotes NGF-induced neurite outgrowth through NF-кB and ERK signaling. In contrast FAIM-L is found only in neurons, where it exerts anti-apoptotic activity against several stimuli. In addition to these two variants, *in silico* studies point to the existence of two additional isoforms, neither of which have been characterized to date. In this regard, here we confirm the presence of these two additional FAIM isoforms in human fetal brain, fetal and adult testes, and placenta tissues. We named them FAIM-S_2a and FAIM-L_2a since they have the same sequence as FAIM-S and FAIM-L, but include exon 2a. PCR and western blot revealed that FAIM-S_2a shows ubiquitous expression in all the tissues and cellular models tested, while FAIM-L_2a is expressed exclusively in tissues of the nervous system. In addition, we found that, when overexpressed in non-neuronal cells, the splicing factor nSR100 induces the expression of the neuronal isoforms, thus identifying it as responsible for the generation of FAIM-L and FAIM-L_2a. Functionally, FAIM-S_2a and FAIM-L_2a increased neurite outgrowth in response to NGF stimulation in a neuronal model. This observation thus, supports the notion that these two isoforms are involved in neuronal differentiation. Furthermore, subcellular fractionation experiments revealed that, in contrast to FAIM-S and FAIM-L, FAIM-S_2a and FAIM-L_2a are able to localize to the nucleus, where they may have additional functions. In summary, here we report on two novel FAIM isoforms that may have relevant roles in the physiology and pathology of the nervous system.

## Introduction

The homeostasis of tissues, organs, and the whole organism is maintained by a fine and continuous balance between cell growth, differentiation, and death. Cell death is, therefore, a fundamental process of life. In this regard, genomes contain several sophisticated cell death-inducing machineries. The regulation of cell death and survival is critical for controlling the number of cells in an organism. Deregulation of these processes can result in pathologies such as neurodegeneration and cancer, conditions characterized by excess of cell death or proliferation, respectively. During development, the removal of excess cells through programmed cell death (PCD) is essential for the establishment and maintenance of the nervous system. Similar regulatory mechanisms that control PCD during development also appear to control PCD in the adult brain[1]. Various proteins regulate these processes and they are organized into two main pathways. The intrinsic pathway is activated by intrinsic signals, such as DNA damage and growth factor starvation. These signals induce the permeabilization of the mitochondrial outer membrane by activating Bcl-2 homology domain 3 (BH3)-only proteins, thus triggering the caspase-9-dependent cascade[2]. In contrast, the extrinsic pathway is activated by death ligands, which bind and activate Death Receptors (DRs) on the cytoplasmic membrane, thereby inducing the recruitment of caspase-8 and/or -10. These initiator caspases trigger apoptosis by activating effector caspases[3]. Various endogenous proteins such as c-FLIP, cIAPs, XIAP, Lifeguard and FAIM are able to inhibit DR-induced apoptosis[4–7].

FAIM molecules are a recently discovered family of evolutionarily conserved proteins structurally unrelated to other DR-induced apoptosis inhibitors[8]. Human *FAIM1* is located in the long arm of chromosome 3 (3q22.3), and it contains six exons and three putative translational start sites in exon 3. To date, two protein products namely FAIM-L and FAIM-S, generated by alternative splicing (AS) have been described[9].

In 1999, FAIM-S was isolated from Fas-resistant B lymphocytes and described as a 20 kDa soluble protein that is ubiquitously expressed and capable of inhibiting Fas-induced cell death [10]. Later, FAIM-S was reported to promote neuronal differentiation and branching through activation of the ERK and NF-kB pathways upon stimulation of nerve growth factor (NGF)[5]. Studies using mouse knock-out of FAIM-L and FAIM-S revealed a phenotype of spontaneous non-hyperphagic obesity accompanied by hepatosteatosis, adipocyte hypertrophy, dyslipidaemia, hyperglycaemia and hyperinsulinaemia[11].

With 66 more nucleotides than FAIM-S, FAIM-L is generated by the inclusion of exon 2b and is expressed exclusively in neurons[9]. Also, FAIM-L has a cytosolic distribution and exerts protection against TNFα- and Fas-induced apoptosis, thereby preventing the activation of caspase 8[12], and/or by interacting with and stabilizing the anti-apoptotic protein XIAP[13]. FAIM-L also acts as a regulator in two neuronal processes that require caspase-3 activation, namely: axon-selective pruning and long-term depression. By stabilizing of XIAP levels and consequent caspase-3 inhibition, FAIM-L prevents these two processes in models of neuronal cells *in vitro*[14]. It has been proposed that loss of FAIM-L function is involved in neurodegenerative diseases such as Alzheimer’s disease (AD). In this regard, FAIM-L levels are reduced in the hippocampus of mouse models of AD (APP/PS1; APP) and in post-mortem human tissue samples and they play a key role in determining the fate of neurons when these cells are exposed to molecules with pro- and anti-inflammatory effects[15].

*In silico* gene expression analysis of human *FAIM1* pointed to the existence of two transcript splice variants of FAIM-S and FAIM-L that include an additional exon. To address this question, here we validate the existence of two new isoforms at the mRNA (both) and protein level (FAIM-S_2a) and show their capacity to modulate neurite outgrowth. Our findings thus, support the notion that these two new variants participate in neuronal differentiation. They also show that FAIM-L and FAIM-L_2a are expressed exclusively in tissues of the nervous system and are regulated by the splicing factor nSR100.

## Materials and methods

### Reagents

Recombinant human sFasL (superFasL, Enzo Life Sciences) and NGF (Sigma-Aldrich, Saint Louis; MO, USA) were used at a concentration of 100 ng/ml. All biochemical reagents were purchased from ThermoFisher Scientific™ (Waltham, MA, USA).

### Human tissue samples

Frozen human samples of fetal brain, fetal and adult testes and placenta were obtained from aborted fetuses at the Pediatric Neurology Unit of the *Hospital Universitari Vall d´Hebron*.

### Ethics statement

The use of tissues from aborted fetuses was approved by the Ethics Committee of the *Hospital Universitari Vall d’Hebron*.

### Cell culture

All cell lines were obtained from the American Type Culture Collection (ATCC, Rockville, MD). Rat pheochromocytoma PC12 cells were grown on collagen-coated 100-mm tissue culture plates (Falcon Discovery Labware; BD Bioscience) in DMEM supplemented with 6% heat-inactivated fetal bovine serum (iFBS) and 6% heat-inactivated horse serum (iHS), 10mM HEPES, 20U/ml penicillin, and 20μg/ml streptomycin. Human embryonic kidney cells (HEK293T), human neuroblastoma cells (SK-N-AS) and kidney epithelial Vero cells (kindly provided by Dr. Albert Pol; IDIBAPS, Hospital Clinic, Barcelona, Spain) were maintained in DMEM supplemented with 10% iFBS, 20U/ml penicillin, and 20μg/ml streptomycin. Human neuroblastoma (SH-SY5Y) cells were grown in DMEM supplemented with 15% iFBS, 20U/ml penicillin, and 20μg/ml streptomycin. Cultures were maintained at 37°C in a humidified atmosphere of 95% air and 5% CO_2_.

### Generation of plasmid constructs

pLDPuro-hsnSR100N lentiviral vector was obtained from Addgene (plasmid #35172) [16]. FAIM-S_2a and FAIM-L_2a expressing vectors were constructed using the coding sequence of human FAIM-S_2a (GenBank^TM^ accession NM_001033030.1) and FAIM-L_2a (GenBank^TM^ accession XM_011512950.2). These sequences were flanked by BamHI/XhoI restriction sites. They were synthesized using the GeneArt system (Thermo Fisher Scientific^TM^), cloned into the pMT4 vector, and subcloned into pcDNA3 containing 3x-FLAG or GFP.

### Cell transfection

HEK293T, PC12, Vero, and SK-N-AS cells at 80% confluence were transfected with the desired expression plasmids using Lipofectamine 2000® (Thermo Fisher Scientific^TM^) in Opti-MEM (Gibco), following the manufacturer’s instructions.

SH-SY5Y cells were transfected using the Amaxa^TM^ 4D-Nucleofector^TM^ (Lonza, Basilea, Switzerland) system. Cell lines were transfected with FAIM isoforms subcloned into pcDNA3containing 3x-FLAG or GFP.

### RNA extraction and RT-PCR

Total RNA was isolated from human cell lines and tissues using the RNeasy Mini Kit (Qiagen) and following the manufacturer´s instructions. Equal amounts of RNA (1 **μ**g) were converted to single-stranded cDNA using the High Capacity RNA-to-cDNA Kit (Applied Biosystems), following the manufacturer’s instructions.

One **μ**l of the resulting cDNA was amplified by PCR using the primers described in Table 1 (Sigma Aldrich).The following PCR conditions were used: 94°C for 3 min, 40 cycles of 94°C for 45 s, 58°C or 55°C for 30 s, 72°C for 1 min and 72°C for 10 min. The ribosomal protein L27 was used as internal control. Experiments were repeated at least three times with independent samples.

**Table 1:** List of specific primers for each exon used for RT-PCR.

Primer Name	Region Amplified	Direction	Sequence 5 -> 3
1aF	*Faim* exon 1a	F	CTTCGGCTAAGGCAGAGGA
1bF1	*Faim* exon 1b	F	GACTACGTCGTGGGATCG
1bF2	*Faim* exon 1b	F	TGGTGAAACCTACCCCAGAG
2aF	*Faim* exon 2a	F	TGGCCCATTCTATCCTATGC
2bF	*Faim* exon 2b	F	GCATCTGGAGATGACAGTCC
2aR	*Faim* exon 2a	R	GCAGAGTCCGGAGATACCAA
2a3R	*Faim* 2a – 3junction	R	TAGGCTGTAAGGAGGGCTCA
2bR	*Faim* exon 2b	R	CCATGGTTGGCAAAAACAGTCTCA
3R	*Faim* exon 3	R	TGCCTGATGTAGTCCCATGT
6R	*Faim* exon 6	R	TTCCGCTTCCCACTACTGAC
nSR100F	*NSR100*	F	ATTGTCGCCAGTATCACGGC
nSR100R	*NSR100*	R	TTTCTTGTGCCTCTTCTCATCTC
L27F	*RPL27*	F	AGCTGTCATCGTGAAGAA
L27R	*RPL27*	R	CTTGGCGATCTTCTTCTTGCC

F: Forward; R: Reverse.

### Western blot analysis

Total protein from the human cell lines was lysed using SET buffer (10 mM Tris-HCl pH 7.4, 150 mM NaCl, 1 mM EDTA and 1% SDS) supplemented with EDTA-free complete protease inhibitor mixture (Roche). Protein from the human tissues was extracted with RIPA buffer (50 mM Tris-HCl pH 7.4–8, 150 mM NaCl, 0.1% SDS, 1% Nonidet P-40 and 0.25% deoxycholic acid), plus 1x complete EDTA-free protease inhibitor (Roche). The suspension was then centrifuged at 16,000 x *g* at 4°C for 30 min, and the supernatants were collected. Protein concentration was quantified by a modified Lowry assay (DC protein assay; Bio- Rad, Hercules, CA). Samples were heat-denatured in loading buffer, resolved by SDS-PAGE and transferred onto polyvinylidene difluoride (PDVF) Immobilon-P membranes (Millipore, Bedford, MA). After blocking with Tris-buffered saline with 0.1% Tween-20 containing 5% non-fat dry milk for 1 h at room temperature, membranes were incubated overnight at 4°C with the following primary antibodies: anti-FAIM (in house antibody [5];1:2000); anti-2b FAIM (in house antibody [5]1:2000); anti-FLAG (Sigma,1:20000); anti-**α**-tubulin (Sigma; 1:20000) and anti-nSR100 (kindly provided by Benjamin Blencowe (Donnelly Centre, University of Toronto, Toronto, ON M5S 3E1, Canada[17]), 1:5000). Horseradish peroxidase-labeled goat anti-rabbit (Sigma, 1:20000) and rabbit anti- mouse IgG (Sigma, 1:20000) were used as secondary antibodies. The following primary antibodies were used for subcellular fractionation: anti-FLAG (Sigma; 1:20000); anti-calnexin (Cell Signaling; 1:1000); anti-actin-HRP (Santa Cruz Biotechnology; 1:1000) and anti-tri-methyl-H3 (Cell Signaling; 1:1000).

Afterwards the membranes were incubated with the chemiluminescent substrate EZ-ECL (Biological Industries, Kibbutz Beit Haemek, Israel). Experiments were repeated three times with independent samples.

### Protein stability assay

PC12 cells were transfected with pcDNA3-FLAG (empty vector), pcDNA3-FLAG-FAIM-L, pcDNA3-FLAG-FAIM-S, pcDNA3-FLAG-FAIM-L-2a or pcDNA3-FLAG-FAIM-S-2a. Four hours after transfection cells were treated with MG-132 (2.5 μM) for 48 h. Cell extracts were lysed using SET buffer and then analyzed by western blot using an anti-FLAG antibody (dilution 1:20000). Anti-tubulin was used as a loading control.

### Subcellular protein fractionation

Subcellular fractionation was performed as previously described[18] with minor modifications. Vero cells were plated at a density of 30,000 cells/cm^2^ and transfected with pcDNA3-FLAG (empty vector), pcDNA3-FLAG-FAIM-L, pcDNA3-FLAG-FAIM-S, pcDNA3-FLAG-FAIM-L-2a or pcDNA3-FLAG-FAIM-S-2a. Cells were homogenized in 10mM HEPES, pH 7.4, 2mM EDTA, 0.32M sucrose and EDTA-free complete protease inhibitor (Roche). Cell homogenates were centrifuged at 600 x *g* for 10 min to remove nuclei (n), then at 3000 x *g* for 10 min to pellet the plasma membrane and heavy intracellular membranes (HM), and finally at 15,000 x *g* for 15 min to yield the light membranes (LM) in the pellet and the microsomal and cytosolic (Cyt) fractions in the supernatant. Supernatants were centrifuged twice at each speed, and pellets were washed twice by resuspension in homogenization buffer and then centrifuged. Pellets were lysed in SET buffer. Protein concentration was quantified by a modified Lowry assay (DC protein assay; Bio- Rad, Hercules, CA). Samples were heat-denatured in Laemmli buffer and subjected to SDS-PAGE. The experiment was repeated twice.

### Immunofluorescence

Vero cells were plated on tissue culture plates (15,000 cells/cm^2^) and transfected with pcDNA3-FAIM-L-2a-GFP or pcDNA3-FAIM-S-2a-GFP. Twenty-four hours later, they were rinsed with PBS 1x at room temperature and fixed with paraformaldehyde 4% for 30 min at room temperature. They were washed then twice with PBS 1x and blocked using 5% bovine serum albumin (Sigma) in PBS1x- 0.1% Triton X-100 for 90 min at room temperature. Cells were incubated overnight at 4°C with anti-calnexin (Abcam; 1:50). Alexa Fluor 594 (ThermoFisher Scientific™; 1:300) was used as a secondary antibody. Mitotracker (ThermoFisher Scientific^TM^; 100nM) was used following the manufacturer’s instructions. Cells were rinsed three times with PBS 1x and incubated with the secondary antibody 1h at room temperature, protected from light. An incubation of 1h at room temperature with a PBS solution containing Hoechst 33258 (Sigma; 0.05 μg/ml) was used for nucleic acid stain. Confocal micrographs were obtained using a FluoView1000 spectral confocal microscope (Olympus). The observations were made in two independent experiments and at least 10 cells per condition were analyzed.

### mRNA stability and real time PCR

SH-SY5Y cells were treated with Actinomycin D (5 μg/ml) for various times (3, 6, 9 and 12 h), to block mRNA synthesis. Total RNA was isolated using the RNeasy Mini Kit (Qiagen) and RT-PCR was performed. Real time PCR (qPCR) amplifications were performed using SybrGreen PCR Master Mix (Applied Biosystems). Samples were run in an ABI Prism 7900HT sequence detector (Applied Biosystems) under the conditions indicated by the manufacturer. Triplicate determinations were averaged at each data-point. 18S amplification was used as an internal control.

### Cell death assays

SK-N-AS cells were plated in 24-well tissue culture plates and transfected with pcDNA3-FLAG (empty vector), pcDNA3-FLAG-FAIM-L, pcDNA3-FLAG-FAIM-S, pcDNA3-FLAG-FAIM-L-2a or pcDNA3-FLAG-FAIM-S-2a. Twenty hours later, they were treated with superFasL. Apoptotic cell death was measured 24 h after treatment. Apoptotic nuclei (condensed or fragmented) were counted after Hoechst 33258 (0.05 μg/ml) staining (1h at room temperature). Experiments were repeated at least three times, and minimum of 500 cells were counted per condition.

### Measurement of neurite outgrowth

PC12 cells were plated in poly-D-Lysine coated tissue culture plates and transfected with pcDNA3-FLAG, pcDNA3-FLAG-FAIM-L, pcDNA3-FLAG-FAIM-S pcDNA3-FLAG-FAIM-L-2a or pcDNA3-FLAG-FAIM-S-2a. Twenty-four hours later, they were treated with NGF (100 ng/ml) for 24h. They were then fixed with 4% paraformaldehyde and photographs of random fields were taken using an inverted microscope (Olympus). Neurite outgrowth was assessed by measuring neurite pixels using the Adobe Photoshop 6.0 software (Adobe Systems, San Jose, CA). Three independent experiments were performed, considering 100 cells per condition.

### *In silico* analyses

GenBank was used to identify the transcripts produced by human *FAIM1*. To predict the secondary structure of FAIM mRNA, we used the Mfold program (version 3.2) http://www.bioinfo.rpi.edu/applications/mfold/ [19] and RNAStructure [20]. Optimal secondary structures for both sequences were obtained in dot-bracket notation with minimum free energy.

### Statistical analysis

Data were analyzed with GraphPad Prism 5 software (GraphPad Software, Inc., La Jolla, CA, USA). Differences in distribution were tested using the t-test or one-way ANOVA. A *p* value less than 0.05 was considered significant.

## Results

### FAIM-S_2a and FAIM-L_2a are novel human FAIM isoforms that include exon 2a

**Fig 1:**
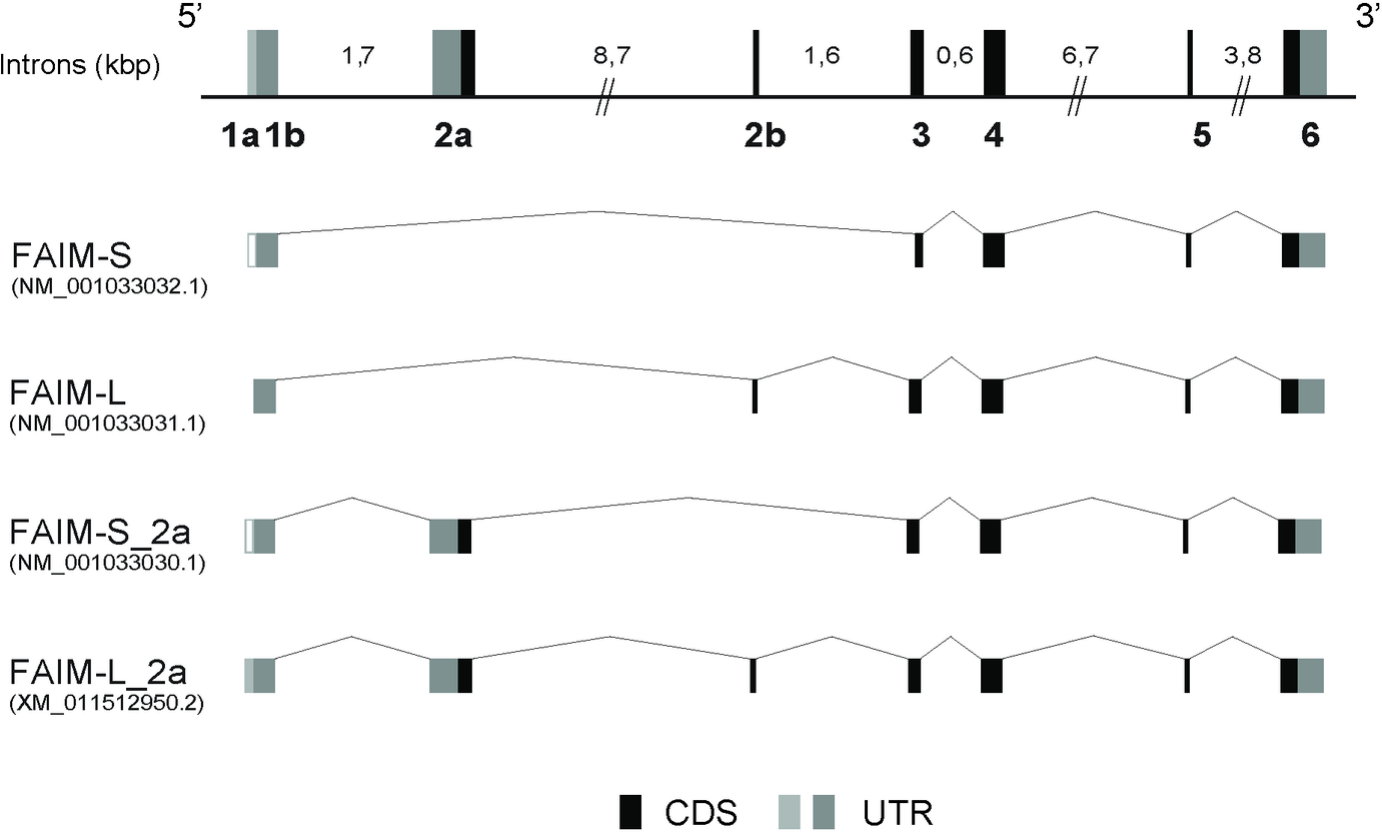
Schematic representation of FAIM isoforms. The genomic structure of *FAIM1* gene is shown. Coding sequences are shown in black boxes (CDS), and non-coding alternative exons in dark gray boxes (UTR), and alternative exons not described in NCBI in white boxes. Intron length is indicated above (kilobase). The new exons described are named following the HUGO nomenclature. The figure shows all transcripts of *FAIM1*. The following four transcripts are represented: FAIM-S (GenBank accession number NM_001033032.1), FAIM-L (GenBank accession number NM_001033031.1), FAIM-S_2a (GenBank accession number NM_001033030.1) and FAIM-L_2a (GenBank accession number XM_011512950.2). CDS: coding sequence; UTR: untranslated region.

I*n silico* analysis pointed to the existence of alternative and uncharacterized FAIM isoforms (GenBank^TM^ accession numbers: NM_001033030.1 and XM_0111512950.2). These isoforms would be generated by the inclusion of exon 2a in the previously described FAIM-S and FAIM-L isoforms (Fig 1).

**Fig 2:**
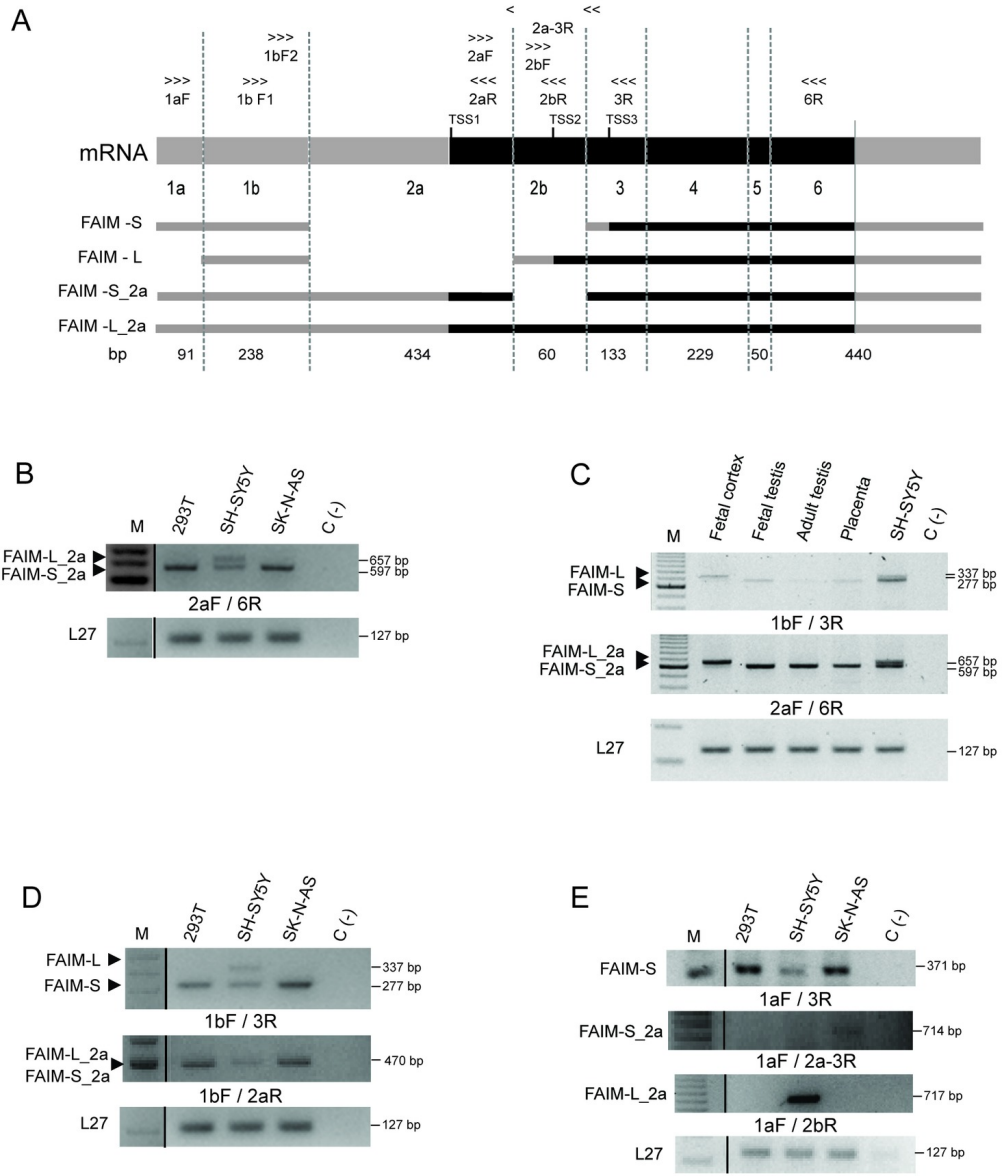
FAIM-S_2a and FAIM-L_2a are expressed in human cell lines and human tissues. **A:** Schematic representation of the primers used for RT-PCR and the localization in each exon. The 5´ and 3´UTRs are represented in gray, and CDS regions in black. Length of exons is indicated in base pairs (bp). TSS: Transcriptional start site, TSS1: TSS of FAIM-S_2a and FAIM-L_2a; TSS2: TSS of FAIM-L; TSS3: TSS of FAIM-S. **B:** RT-PCR analysis of exon 2a in human cell lines using a 2aF/6R primer combination (the 2aF primer is common to exon 2a in FAIM-L_2a and FAIM-S_2a). Bands of 657 and 597 bp were detected in the neuroblastoma cell line (SH-SY5Y) corresponding to the predicted size of FAIM-L_2a and FAIM-S_2a, respectively. In non-neuronal derived cell lines, namely HEK293T and SK-N-AS, only one band of FAIM-S_2a was detected. **C:** RT-PCR analysis of all the four isoforms in the fetal cortex, fetal and adult testes and placenta using specific primers for exon 2a (2aF/6R) and 1b (1bF/3R). **D:** RT-PCR analysis of exon 1b in HEK293T, SH-SY5Y and SK-N-AS cell lines. Primers used: 1bF/3R or 1bF/2aR. Only one band was observed in SH-SY5Y cells, but FAIM-L_2a and FAIM-S_2a were estimated to have a band of 470 bp. **E:** mRNA amplification of FAIM-S, FAIM-S_2a and FAIM-L_2a using a primer in the region of exon 1a (1aF/3R; 1aF/2a-3R; 1aF/2bR). Negative control (C(-)) was performed with water instead of cDNA. 100 bp DNA Ladder Plus was used to determine the size of each DNA band. L27 was used as an internal control. The primers used are indicated below the agarose gel.

We sought to first confirm the existence of these additional transcripts at the mRNA level. To this end, we designed specific primers for each *FAIM1* exon using the sequences reported in GenBank (Fig 2A) in order to perform RT-PCR using cDNA isolated from human neuronal and non-neuronal cells (SH-SY5Y, SK-N-AS, and HEK293T) and human tissue samples. While FAIM-S_2a was detected in all the human cell lines, FAIM-L_2a was detected only in the neuronal cell line SH-SY5Y (Fig 2B). The SK-N-AS cell line was included as a control of neuronal-lineage but with no detectable expression of FAIM-L. Moreover, tissue samples analyses showed that FAIM-L_2a mRNA was present only in the fetal cortex, while FAIM-S_2a was detected in all the tissues tested (Fig 2C).

#### Both exon 1a and 1b are present in FAIM isoforms

The GenBank sequences of the four isoforms (NM_001033032.1, NM_001033031.1, NM_001033030 and XM_0011512950.2) shared exon 1b. However *in silico* tools, predicted the FAIM-L_2a sequence to contain 91 additional nucleotides at the 5’UTR (exon 1a, light gray square in FAIM-L_2a in Fig 1). To ascertain the structure of exon 1 in the isoforms, we mapped it by RT-PCR. We used specific primers to amplify exon 1a and exon 1b (Fig 2A and Table 1) in human cell lines. Exon 1b was detected in the four isoforms (Fig 2D). FAIM-S and FAIM-S_2a were amplified in all the cell lines with products of 277 bp for FAIM-S and 470 bp for FAIM-S-2a. FAIM-L (337 bps) and FAIM-L_2a (470 bps) were amplified only in SH-SY5Y cells. Primers used in the exon 1b region were unable to distinguish between FAIM-S_2a and FAIM-L_2a since they showed the same sequence length. The inclusion of exon 1b in the two isoforms was confirmed by product size using another primer combination. These results suggest that the 5´UTR of all the isoforms contain exon 1b thereby confirming what is reported in GenBank (Fig 2A and 2D).

We then performed RT-PCR analysis using specific primers for exon 1a in order to determine the presence of this region in the sequences of the isoforms (Table 1). Three of the four transcripts were amplified using a forward primer in exon 1a (1aF). We found that FAIM-S (expressed in all cell lines) with the expected size 371 bp, FAIM-S_2a (only detected in SK-N-AS) with the expected size 714 bp, and FAIM-L_2a (expressed in SH-SY5Y) with the expected size 717 bp contained exon 1a in the 5´UTR (Fig 2E).

### Similar mRNA stability in FAIM isoforms

**Fig 3:**
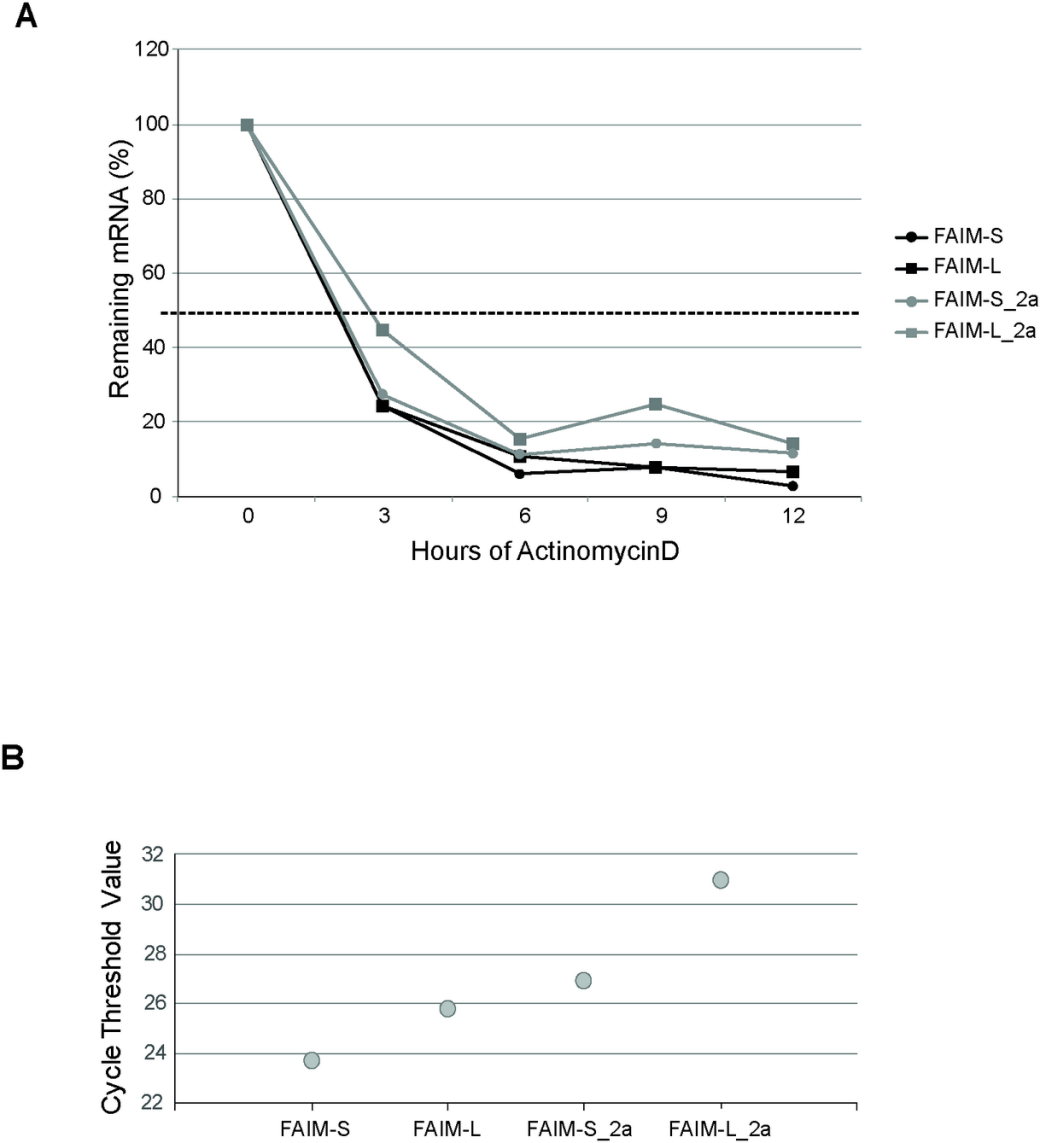
Treatment with Actinomycin D (ActD). SH-SY5Y cells were treated with ActD for a range of times (3, 6, 9 and 12 h). **A:** The half-life of mRNA was measured by treating cells with ActD (5 μg/ml) and collecting total RNA at the times shown, whereupon the levels of *FAIM* mRNA and *18S mRNA* (a stable, housekeeping control mRNA) were measured by RT–qPCR analysis. mRNA half-life was calculated as the time needed to reduce transcript levels to half (50%, discontinuous line) of their initial abundance at time 0. **B:** Number of cycles needed to detect similar size product of FAIM-S, FAIM-L, FAIM-S_2a and FAIM-L_2a by qPCR (SybrGreen) using the following pairs of primers: (1bF2/3R for FAIM-S; 2bF/3R for FAIM-L; 2aF/2a3R for FAIM-S_2a and 2aF/2bR for FAIM-L_2a).

To better understand the mRNA structure of these isoforms, we studied mRNA stability. SH-SY5Y cells were treated with the inhibitor of transcription Actinomycin D (5 **μ**g/ml) for 3, 6, 9 and 12h. At different time points, RNA was isolated and qPCR was performed. mRNA half-life was calculated as the time needed to reduce transcript levels to half (50%, discontinuous line) of their initial abundance at time 0. The half-life of all the isoforms was around 165–180 min (Fig 3A). Following the study, differences in the abundance of mRNA transcripts in SH-SY5Y cells were found. The number of cycles to amplify (similar in size product) FAIM-S, FAIM-L, FAIM-S_2a and FAIM-L_2a by qPCR was different for each isoform, thereby indicating differences in its abundance in SH-SY5Y cells. FAIM isoforms were detected at 24 cycles for FAIM-S, 25 cycles for FAIM-L, 27 and 31 cycles for FAIM-S_2 and FAIM-L_2a respectively. These findings reveal a higher presence of FAIM-S mRNA and lower levels of FAIM-L_2a (Fig 3B).

### FAIM-S_2a is consistently translated to protein

**Fig 4:**
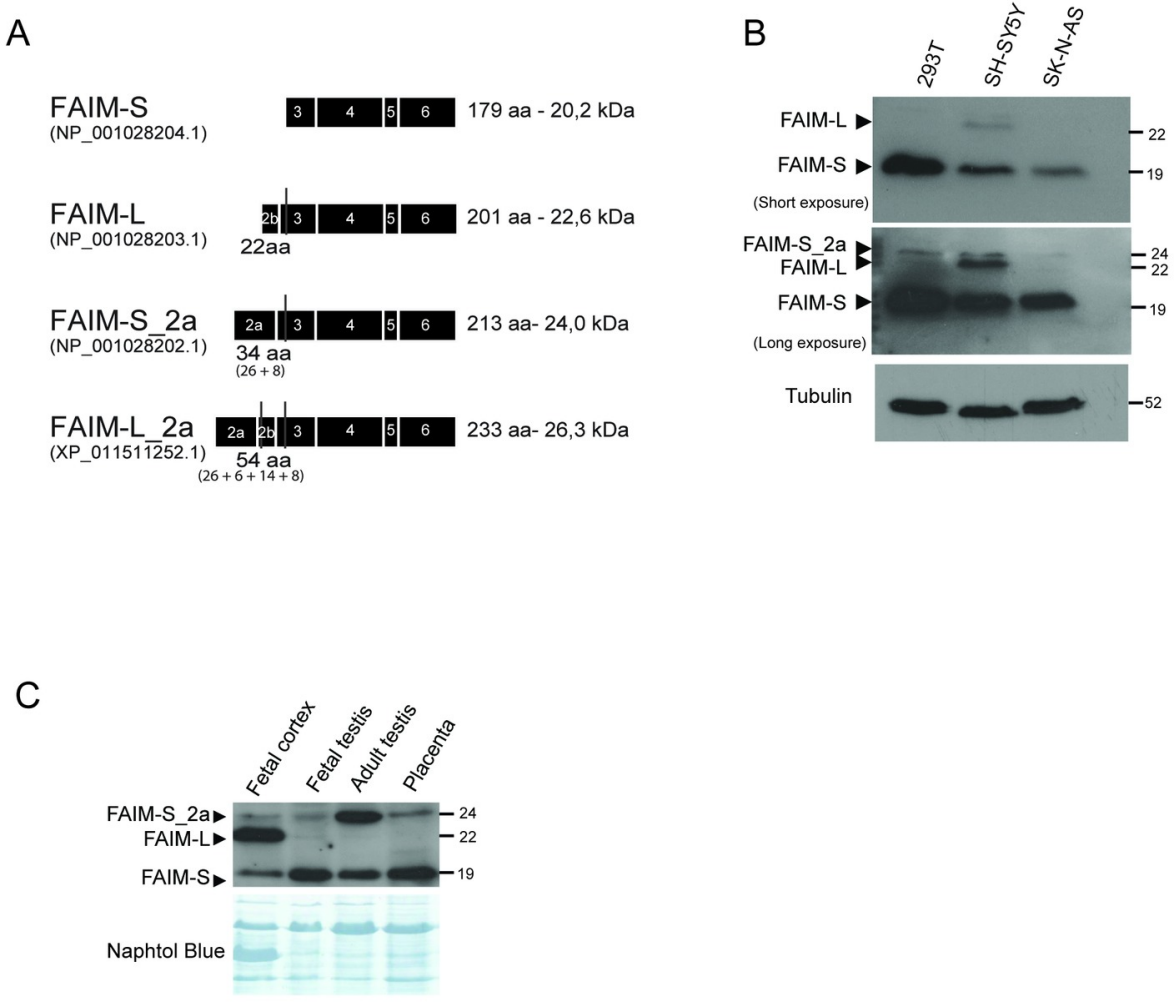
The protein FAIM-S_2a is expressed in all the human cell lines and tissues analyzed. **A:** Schematic representation of predicted protein structure of each isoform. FAIM-S (GenBank: NP_001028204.1) has a predicted molecular weight of 20.2 kDa and 179 amino acids (aa). FAIM-L (GenBank: NP_001028203.1) has an apparent molecular weight of 22.6 KDa with 22 additional residues at the N-terminus compared to FAIM-S. FAIM-S_2a (GenBank: NP_001028202.1) has an extra 34 aa in the N-terminus and FAIM-L_2a contains an extra 22 aa of FAIM-L plus 32 aa (GenBank: XP_011511252.1). **B:** Western blot analysis of the human cell lines (HEK293T, SH-SY5Y and SK-N-AS) using anti-FAIM antibody (dilution 1:2000). Two different exposures of the film are shown in order to facilitate observation of the bands of all isoforms. Anti-tubulin was used as a loading control. **C:** Western blot analysis of fetal cortex, fetal and adult testes, and placenta. An anti-FAIM antibody was used to detect FAIM expression in human tissues. Naphtol blue staining was used as a loading control.

We next examined whether FAIM-S_2a and FAIM-L_2a are expressed at the protein level in human cell lines and human tissues. Using the ProtParam tool available at expasy.org, we predicted the molecular weight of each isoform, indicated in Fig 4A. Western blot analysis of HEK293T, SH-SY5Y and SK-N-AS cell lines showed a band of 20 kDa corresponding to FAIM-S and a band of 24 kDa compatible with the apparent and predicted molecular weight of FAIM-S_2a in all cell lines. This finding suggested that FAIM-S_2a is expressed ubiquitously, similarly to FAIM-S. A band of 23 kDa compatible with the predicted molecular weight of FAIM-L was detected only in the neuronal SH-SY5Y cell line, but FAIM-L_2a expression was not detected. When the analysis was performed in human tissues, only FAIM-S_2a was detected in fetal cortex, fetal and adult testes and placenta (Fig 4C). FAIM-L_2a was not detected in any sample, suggesting that this isoform was either below detectable levels or not translated to protein.

**Fig 5:**
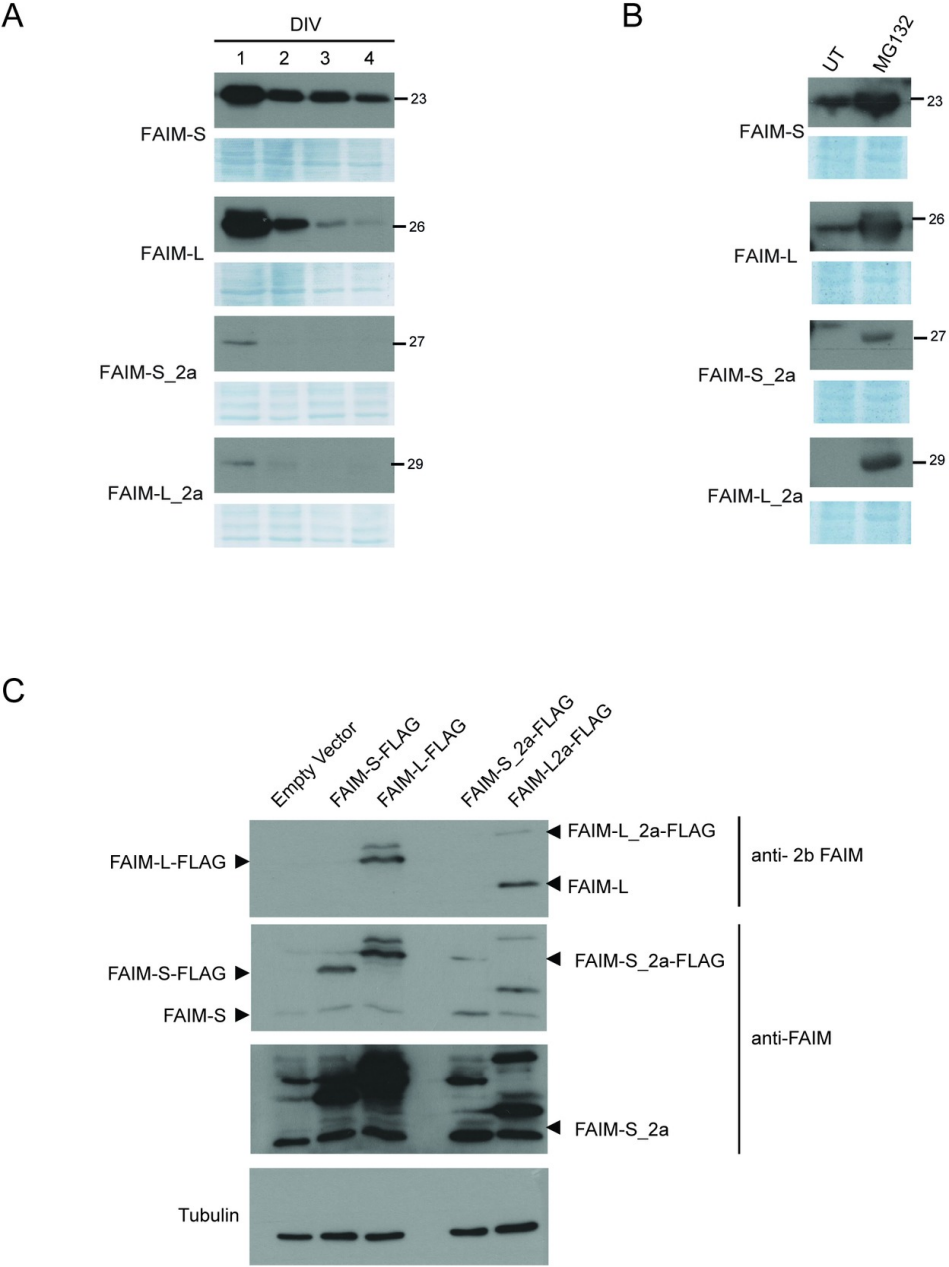
Isoforms expression in cell lines. **A:** SH-SY5Y cells were transfected with the pCDNA3-FLAG-FAIM-S, pCDNA3-FLAG-FAIM-S_2a, pCDNA3-FLAG-FAIM-L or pCDNA3-FLAG-FAIM-L_2a vector. At a range of time points, cells were harvested and protein expression was assessed by western blot using an anti-FLAG antibody (dilution 1:20000). **B**: PC12 cells were transfected with the isoform vectors (above mentioned) and treated with MG-132 (2.5 μM). Cell extracts were then resolved by western blot analysis, and FAIM expression was measured using an anti-FLAG antibody (dilution 1:20000). **C:** HEK293T cells transfected with pcDNA3-FLAG-FAIM-L, pcDNA3-FLAG-FAIM-S, pcDNA3-FLAG-FAIM-L-2a or pcDNA3-FLAG-FAIM-S-2a vector were lysed, and protein extracts were analyzed by western blot. An anti-FAIM-L (anti-2b FAIM, specific for neuronal exon 2b) and anti-FAIM (that recognizes the common part of the isoforms) were used. Anti-tubulin was used as a loading control. Two different exposures of the film are shown in order to facilitate observation of the bands of all isoforms. DIV: days *in vitro* (n = 3).

Furthermore, we observed that FAIM-L_2a and FAIM-S_2a showed only slight overexpression in comparison with overexpression of the shorter isoforms (i.e. FAIM-L and FAIM-S). Given the lack of detectable endogenous FAIM-L_2a protein and the low levels of endogenous FAIM-S_2a, we sought to characterize the two proteins in transient transfection conditions, in order to have a consistent amount of both proteins. After transfection, the expression of the extra-long isoforms dropped drastically compared to the transfected forms of FAIM-L and FAIM-S, which both maintained their expression over several days in cell culture (Fig 5A). Experiments using the proteasome inhibitor MG-132 (Fig 5B) showed accumulation of FAIM-L_2a and FAIM-S_2a after 48h of culture, thereby suggesting that these proteins were partially and rapidly degraded via the proteasome. When FAIM-L_2a and FAIM-S_2a were overexpressed, we also observed an increase in the shorter corresponding isoforms of FAIM-L and FAIM-S, respectively. Using an antibody specific for the neuronal exon 2b, we found that FAIM-L was detectable in HEK293T cells when FAIM-L_2a was overexpressed (Fig 5C). This result suggests that the translational start site of FAIM-S and FAIM-L is preferential or that the longer forms give rise to the shorter ones through proteolysis. Using a dot blot technique, we discarded the proteolytic hypothesis, since the FLAG at the N-terminal of FAIM-L_2a was not present in the lysate. (S1 Fig).

### FAIM-S_2a and FAIM-L_2a mRNA secondary structures have lower levels of thermodynamic stability

To better understand the absence of FAIM-L_2a protein expression, we analyzed the 5´UTR in *FAIM1*. It is known that a widespread mechanism modulating mRNA translational efficiency depends on short upstream open reading frames (uORFs) encoded in sequence of this region[21]. uORFs tend to reduce the translation efficiency of downstream protein-coding ORFs[22–24]. uORFs are found in up to 50% of mammalian genes and their utilization is frequently regulated by alternative splicing[25,26].

The transcriptional start site (TSS) for FAIM-L_2a and FAIM-S_2a is located in exon 2a, for FAIM-L in the exon 2b and for FAIM-S in the exon 3. To study the differential regulation of translation, we used bioinformatics tools to study the sequence of the 5’ UTRs of these isoforms to identify potential regulatory elements, such as uAUGs, mRNA secondary structures and G/C rich sequences that may impede translation. The 5’UTR sequences of FAIM-S_2a and FAIM-L_2a included exons 1a, 1b and the sequence of exon 2a that precedes the TSS. The two extra-long isoforms therefore share the same 5’UTR sequence of 683 nucleotides. On the other hand, FAIM-S and FAIM-L have shorter 5’UTR sequences, with 329 nucleotides (FAIM-S 5’UTR includes exon 1a, 1b and the exon 3 sequence that precedes its TSS) and 238 nucleotides (FAIM-L 5’UTR includes exon 1b and the exon 2b sequence that precedes the TSS), respectively. Using ORFfinder (www.ncbi.nlm.nih.gov/orffinder/), we observed that FAIM-S_2a and FAIM-L_2a contain 3 uAUGs in the sequence of exon 2a included in the 5’UTR, thereby pointing to a more complex regulation of these longer isoforms.

**Fig 6:**
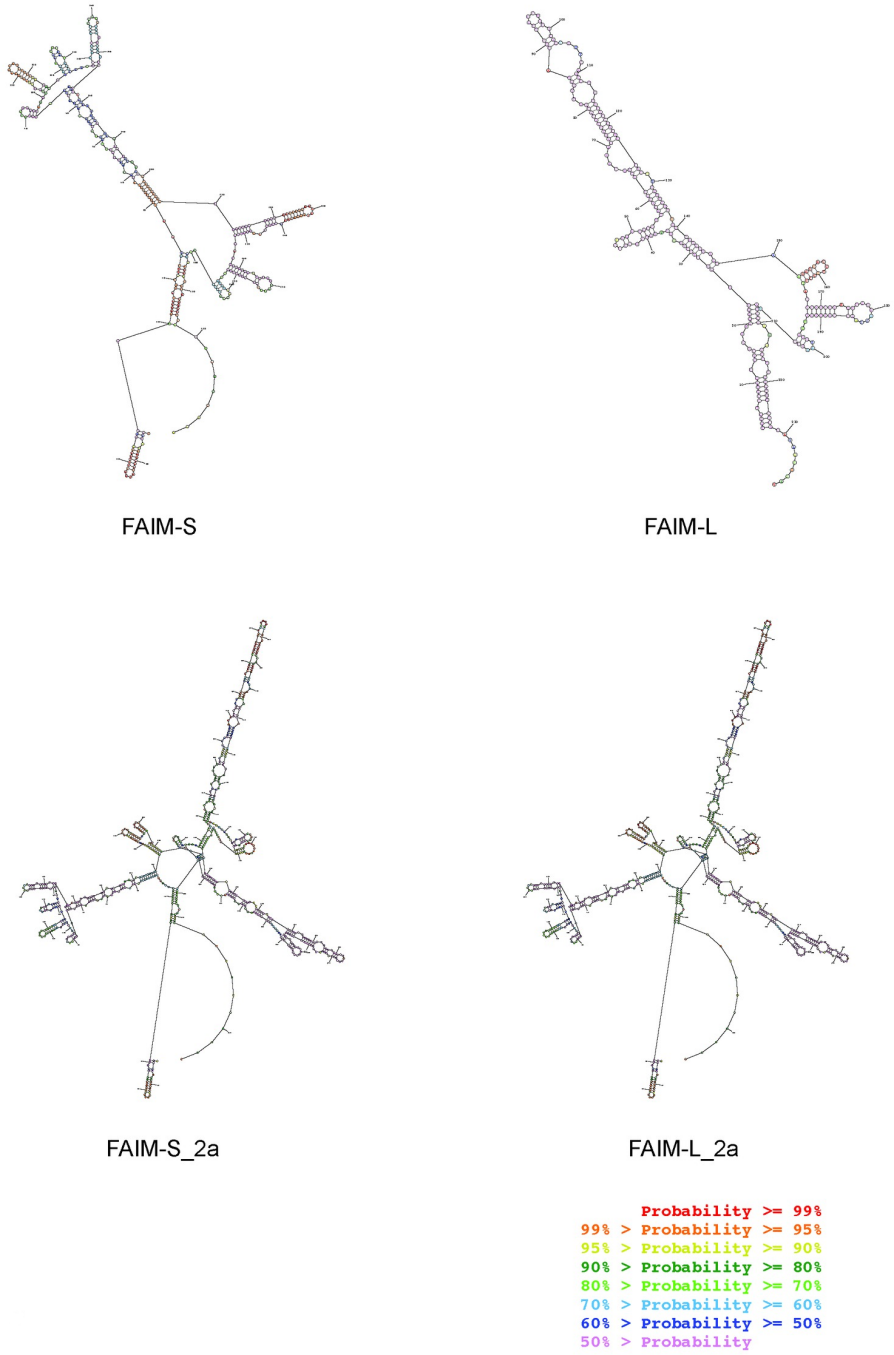
mRNA secondary structure. 5´UTR sequences of FAIM isoforms structures as shown by the output of the RNAStructure web server (http://rna.urmc.rochester.edu/RNAstructureWeb/Servers/Predict1/Predict1.html). The optimal secondary prediction for all the sequences was obtained in dot-bracket notation with the lowest free energy structure for the input sequence. Colour annotation of the structures provides information about the confidence in the prediction of a specific pair (base paired or unpaired nucleotides). The highest probabilities are red and the lowest are purple.

Another decisive element in the regulation of mRNA translation is the secondary structure of the 5´UTR[19]. We used the MFold and RNAStructure web servers to predict and analyze 5’UTR structure of the four isoforms. A higher level of thermodynamic stability and a higher presence of Guanine/Cytosine-rich sequences (G/C) for the secondary structure of FAIM-S_2a (dG = -279.4 Kcal/mol, with 45.9% G/C) and FAIM-L_2a (dG = -279.4 Kcal/mol, with 45.8%G/C) was detected compared to FAIM-S (dG = -156.9 Kcal/mol, with 43.7% G/C) or FAIM-L (dG = -110.9 Kcal/mol, with 41.8% G/C) respectively (Table 2) (Fig 6). A lower Gibb’s free energy (dG) indicates stronger secondary RNA structure formation and a lower probability of translation. Therefore, the difference in dG values may explain the increased complexity of the translation of these two extra-long isoforms and their lower expression levels.

**Table 2:** Thermodynamic stability of the secondary structure of the 5´UTR.

Gene	Region	dG (Kcal/mol)	5´UTR Length	% G/C
***FAIM-S_2a***	5' UTR	-279.4	683 nt	45,9
***FAIM-L_2a***	5' UTR	-279.4	683 nt	45,8
***FAIM-L***	5' UTR	-110.9	238 nt	41,8
***FAIM-S***	5' UTR	-156.9	329 nt	43,7

Undetectable levels of FAIM-L_2a in human tissues may also be due to a different temporal regulation. Here we analyzed the expression of FAIM-L_2a protein only in the fetal cortex and therefore we cannot rule out the possibility that this isoform is expressed in adult brain or under pathological conditions.

### Alternative splicing of exon 2b is regulated by nSR100 in FAIM-L and FAIM-L_2a

**Fig 7:**
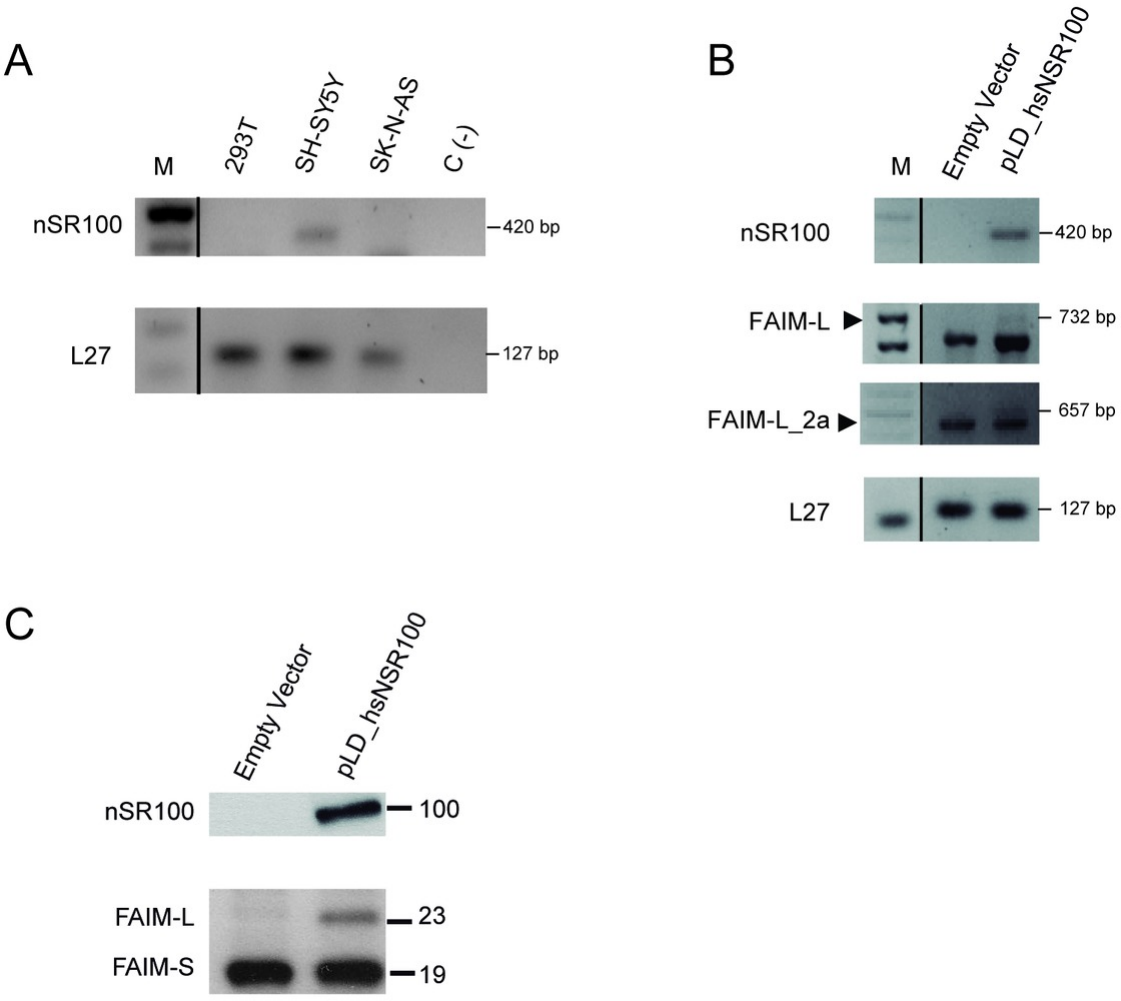
Overexpression of nSR100 induce FAIM-L_2a and FAIM-L in HEK293T cells. **A:** nSR100 transcript was amplified by RT-PCR. In SH-SY5Y cells, a band of 420 bp was detected. **B:** RT-PCR in HEK293T cells after transient transfection with nSR100 using Lipofectamine 2000®. Transcripts of FAIM-L and FAIM-L_2a were observed at 732 bp and 657 bp bands. L27 was used as an internal control in all PCRs. **C:** Western blot analysis using anti-FAIM in HEK293T cells after transfection with nSR100 vector (pLD_hsnSR100). A band of 23 kDa (FAIM-L) was detected in nSR100 transfection conditions. As a negative control, we used an empty vector (n = 3).

The reason why FAIM-L isoforms are restricted to neuronal tissues is unknown. A recent study reported that neuron-specific splicing isoforms are regulated by the 100-kDa Neural-Specific Serine/Arginine Repetitive Splicing Factor (nSR100)[27,28]. To confirm the role of nSR100 in the expression of FAIM-L isoforms, we examined its expression in various cell lines (HEK293T, SH-SY5Y and SK-N-AS). nSR100 was expressed only in the human neuronal SH-SY5Y cell line, where it showed the same expression pattern as that of FAIM-L (Fig 7A). To confirm the regulation of FAIM-L and FAIM-L_2a by nSR100, we ectopically expressed nSR100 in the non-neuronal HEK293T cell line and analyzed FAIM-L isoforms expression by RT-PCR. nSR100 overexpression in HEK293T cells induced mRNA expression of FAIM-L and FAIM-L_2a (Fig 7B). When protein levels were analyzed in the same conditions, only a band of 23 kDa, corresponding to FAIM-L, was detected (Fig 7C). Expression of FAIM-L_2a remained below detection levels. These results indicate that nSR100 promotes the expression of the two FAIM-L isoforms through the inclusion of exon 2b.

### FAIM-S_2a and FAIM-L_2a have a cytosolic and nuclear distribution pattern

**Fig 8:**
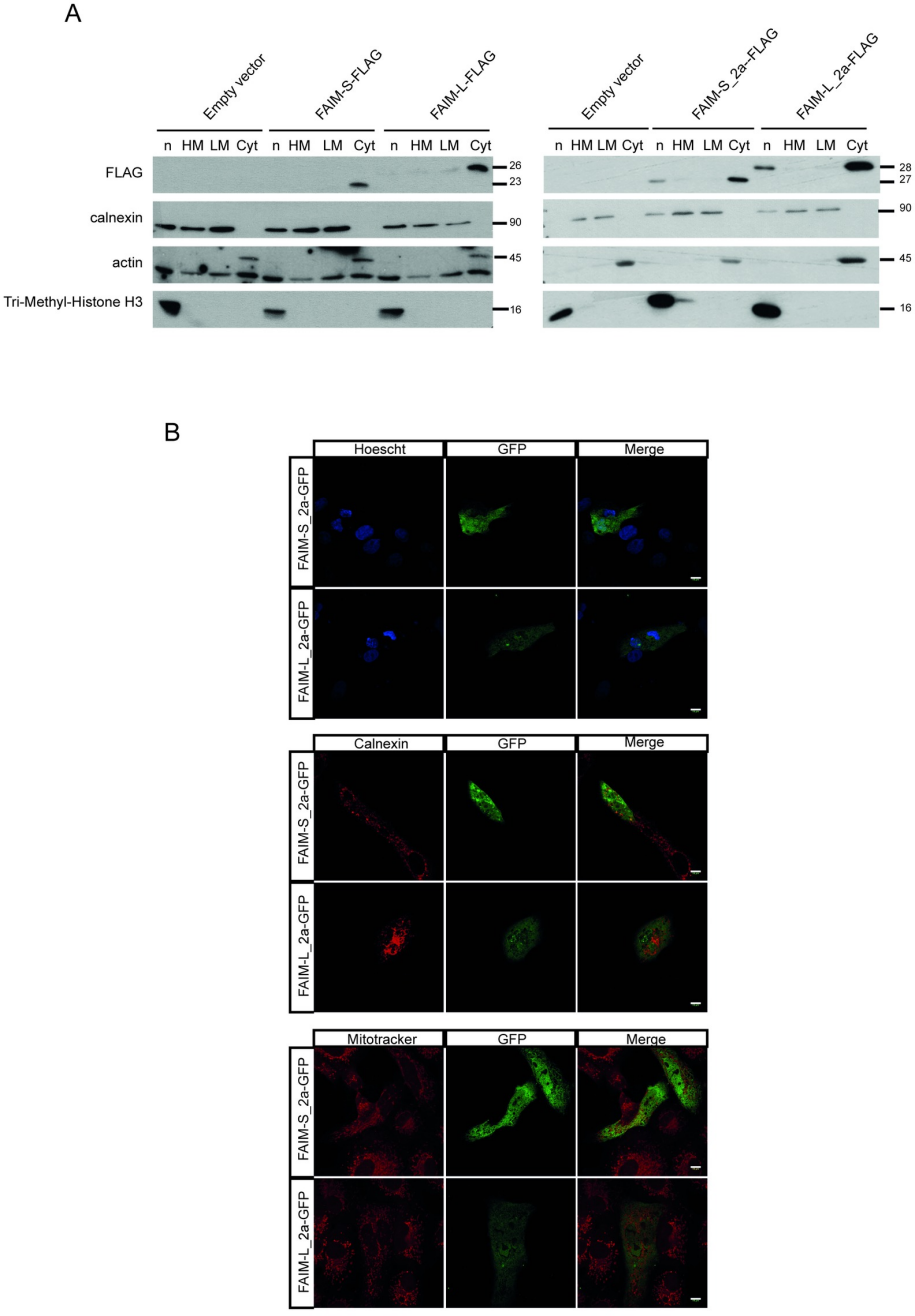
FAIM-S_2a and FAIM-L_2a are localized in the cytoplasm and nucleus. **A:** Western blot analysis using anti-FLAG to detect the presence of FAIM-S, FAIM-L, FAIM-S_2a and FAIM-L_2a in the distinct cellular compartments. Anti-calnexin was used as a marker for the membrane fraction, anti-actin as a marker of the cytosolic fraction, and anti-Tri-Methyl-Histone H3 as a marker of the nucleus. **B:** Immunofluorescence in Vero cells 24 h after transfection with pcDNA3-GFP containing the extra-long isoforms. Anti-calnexin (reticular protein), Mitotracker (mitochondrial marker) and Hoechst (nuclei staining) were used to examine the co-localization of FAIM isoforms. Scale bars 10 **μ**m.

FAIM-L and FAIM-S are reported to be cytosolic soluble proteins showing a diffuse cytosolic pattern that excluded the nucleus[10,12]. To study the localization of the new isoforms, we performed subcellular protein fractionation in cells ectopically expressing FAIM-S_2a and FAIM-L_2a. Unexpectedly, these two isoforms were detected in the nuclear and cytosolic fractions (Fig 8A), thereby suggesting that they serve a potential role in the nucleus. Immunocytochemical analysis confirmed the presence of FAIM-S_2a and FAIM-L_2a in the nucleus and in the cytosol (Fig 8B).

### FAIM-S_2a and FAIM-L_2a increase neurite length in NGF-stimulated PC12 cells

**Fig 9:**
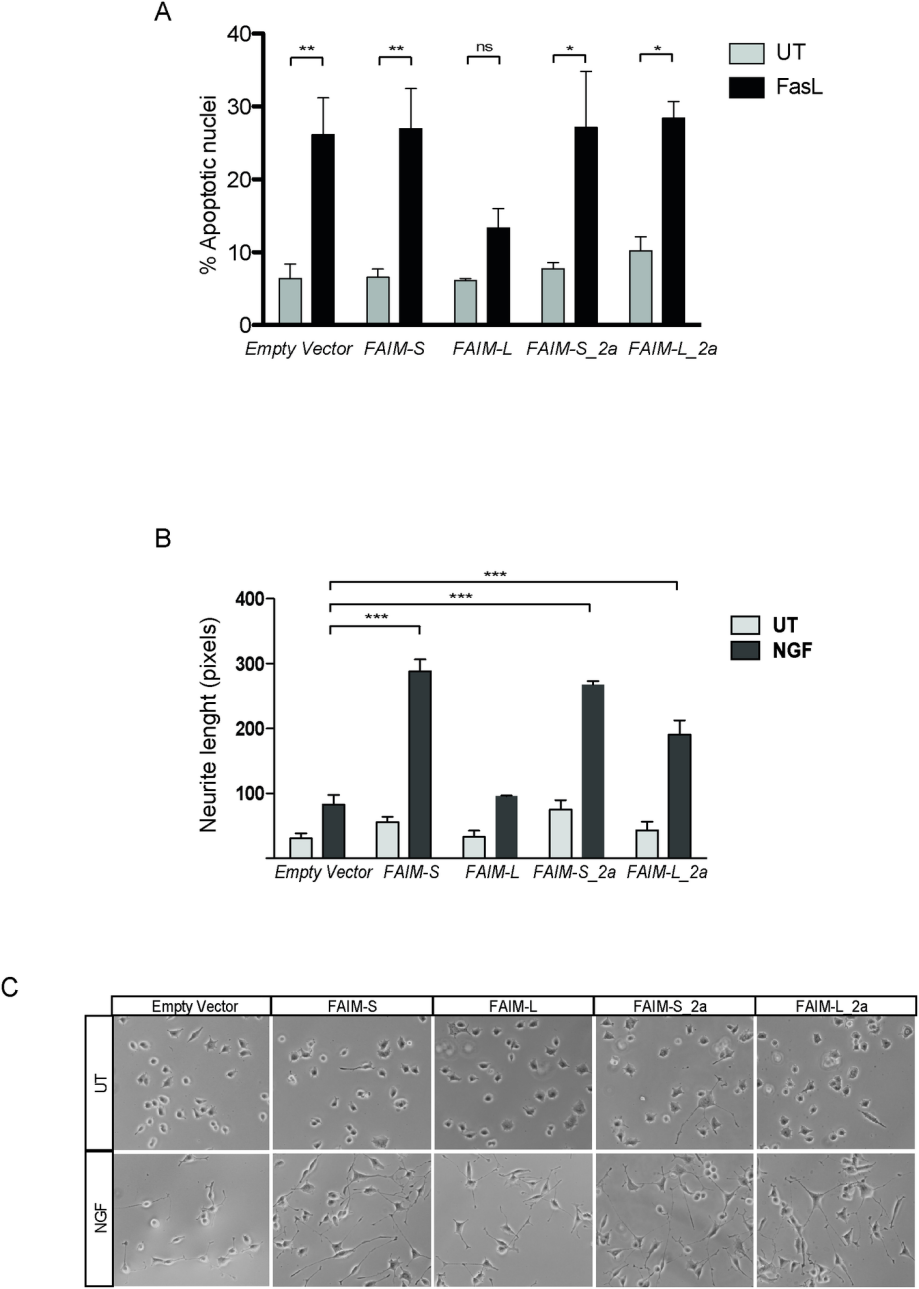
FAIM-S_2a and FAIM-L_2a increase NGF induced neurite outgrowth in PC12 cells. **A:** Cell death was measured in SK-N-AS cells by counting condensed nuclei after Hoechst 33258 (0.05 μg/ml) staining. Cells were transfected with empty vector (pcDNA3-FLAG) or pcDNA3-FLAG-containing all the isoforms and then treated or not with FasL (100 ng/ml). Graph represents the percentage of apoptotic nuclei of a minimum of 500 cells per condition. Experiments were repeated at least three times. Statistical significance was determined by one-way ANOVA. *ns*: no significant and *p<0.05;**p<0.01. **B:** Neurite length was measured in PC12 cells treated with NGF (100ng/ml) or left untreated after transfection of the isoform vectors. At least three independent experiments were performed. Statistical significance was determined by one-way ANOVA, ***p<0.001. **C:** Representative images of neurite outgrowth in the different conditions. UT: untreated; NGF: nerve growth factor (100 ng/ml) (n = 3 per experiment).

To assess an initial functional role of these new isoforms, we compared the function of FAIM-L_2a and FAIM-S_2a in cell differentiation and resistance to DR-induced cell death, properties previously attributed to FAIM-S and FAIM-L respectively (reviewed in[8]). We transfected the FLAG-tagged forms of FAIM-L, FAIM-S, FAIM-L-2a, and FAIM-S-2a into neuroblastoma derived cells and stimulated these cells with soluble Fas Ligand (sFasL). FAIM-L was the only protein that reduced FasL-induced apoptosis (Fig 9A). Next, we studied the modulation of neurite outgrowth after NGF stimulation. All isoforms, except FAIM-L, increased the neurite length of neuronal cells stimulated with NGF (Fig 9B). This result suggests that FAIM-S-2a and FAIM-L_2a participate in neurite outgrowth rather than in the prevention of DR-induced cell death.

## Discussion

In recent years, the application of genome-wide profiling technologies, coupled to bioinformatics approaches has revealed that more than 90% of human genes undergo alternative splicing[29,30]. This process is crucial for regulating the levels and tissue specificity of gene expression, and any disruption of this mechanism can lead to disease[30,31]. Eukaryotic gene expression is regulated at the level of mRNA translation and stability, in which regulation of the 5´UTR plays a fundamental role[32]. In the present study, we focused on the characterization of splicing products of the human *FAIM1*, a death receptor antagonist known to participate in neuronal differentiation, DR-induced apoptosis, obesity and hyperinsulinaemia[11]. The two isoforms of *FAIM1* first described were FAIM-S and FAIM-L. In this regard, FAIM-S was first reported in 1999 as an antagonist of Fas in lymphocytes[10]. It promotes neuronal differentiation by NGF activating ERK and NF-**κ**B pathways[5]. FAIM-L, a longer splice variant that contains 22 additional amino acids at the N-terminal end of the protein was identified in 2001[9]. This isoform protects neurons from DR-induced cell death by either binding to the Fas receptor and preventing the activation of caspase-8, thereby inhibiting the apoptotic cascade at initiator caspase level[12], or by stabilizing the antiapoptotic protein XIAP[13]. In addition to these two previously known isoforms, two longer splicing isoforms (FAIM-S_2a and FAIM-L_2a) are reported in databases; however, this is the first study to provide details of their expression and function. Here we characterized FAIM-S_2a and FAIM-L_2a and the role of these isoforms in neurite outgrowth and cell death.

Studies by Zhong and col. suggested that FAIM-S and FAIM-L isoforms are generated by AS[9].This process is crucial in the evolution of increased proteomic and functional complexity and is especially relevant in the nervous system[33]. FAIM-L mRNA expression increases during development, while that of FAIM-S remains unaltered[12].

AS has played a major role in the evolutionary expansion of the proteomic and functional complexity underlying many cellular processes and is especially prevalent in the vertebrate nervous system[34,35]. The mechanisms that control neural-specific AS are poorly understood. AS is regulated by a member of a large class of proteins harboring Ser/Arg (SR)-repetitive regions[36]. The vertebrate- and neural-specific SR-related protein of 100 kDa (nSR100/SRRM4) regulates a network of brain-specific alternative exons concentrated in genes involved in various aspects of neurogenesis[16,27]. nSR100 is required for neural cell differentiation *in vivo*. Knockdown of nSR100 disrupts the inclusion of a large set of nervous system specific alternative exons[27]. In fact, our results show that the ectopic expression of nSR100 in non-neuronal cells (i.e. HEK293T) induced the formation of the neuronal-specific splice variants FAIM-L and FAIM-L_2a. Concurring with our observation, FAIM-L also appeared in a list of genes modulated by nSR100 in HEK293T cells[28]. The expression of this protein may be the factor that regulates the inclusion of exon 2b, and therefore the nervous system specific expression pattern of FAIM-L and FAIM-L_2a. In fact, there is a putative nSR100 binding site before the start of exon 2b (-41 GCTGC/GCTGCT -13).

All the isoforms examined contained exon 1b (238 nucleotides). In this study, we confirm that exon 1a (91 nucleotides) is also present in FAIM-L_2a, FAIM-S_2a and FAIM-S, thereby indicating that the previous annotated sequences for FAIM-S (NM_001033032.1) and FAIM-S_2a (NM_001033030.1) are not accurate. In summary, all the FAIM isoforms examined except FAIM-L included exon 1a.

While we detected FAIM-L_2a mRNA in human cell lines and tissues, we were unable to detect its protein expression. Fig 5C and S1 Fig show that, in presence of FAIM-L_2a overexpression, most overexpressed mRNA is translated to FAIM-L, as reflected by an induction in FAIM-L expression. Since the TSS of FAIM-L and FAIM-S are conserved in the mRNA sequences of the longer isoforms (Fig 2A), we hypothesise that the increase in FAIM-L expression is attributable to the TSS of FAIM-L being preferential.

Moreover, as we show in Fig 6 and Table 2, the thermodynamic stability of the 5’UTR secondary structure of FAIM-L_2a is lower than that of FAIM-L, thereby indicating differences in mRNA stability. The secondary structure complexity of FAIM-L_2a mRNA may hinder the binding of the ribosomal complex and therefore its translation into protein.

FAIM-S_2a was less expressed in fetal tissues and placenta; we should therefore consider that the low levels of the isoforms may be due to differential temporal expression. The presence of CpG islets in the 5´UTR of *FAIM1* may point out to a role in the adult, since it has been demonstrated that such regions can define differential pre- or postnatal expression.

The analysis of protein localization shows that FAIM-L_2a and FAIM-S_2a overexpression localized in the cytoplasm and nucleus. In contrast, FAIM-L and FAIM-S were located only in the cytoplasm. Supporting the localization of both isoforms (FAIM-L_2a and FAIM-S_2a) in the nucleus, we found the presence of a nuclear export signal (NES), in the amino acid sequence encoded by exon 2a. NESs are usually found in proteins with nuclear functions and they facilitate active transport through the nuclear membrane[37]. The role of these isoforms in the nucleus is unclear and further experiments are required to elucidate this question.

While FAIM-S_2a has similar functions to FAIM-S in promoting neurite outgrowth, FAIM-L_2a was not capable of recapitulating FAIM-L function in preventing DR-induced cell death. One possible explanation may be that the first 22 amino acids of FAIM-L are essential for interaction with XIAP[13]. Since the FAIM-L_2a sequence has 26 additional amino acids at the N-terminal of the protein, the known interactions with FAIM-L partners could be lost.

In summary, here we provide the first evidence of FAIM-S_2a and FAIM-L_2a expression in human cell lines and tissues suggesting that these two isoforms could play a significant role in the nervous system.

## Supporting information

S1 Fig FAIM-S_2a and FAIM-L_2a mRNA can be processed to FAIM-S and FAIM-L. HEK293T cells were transiently transfected with the four FLAG-tagged isoforms (Fig 5C). 2.5μg of lysate was spotted on a Nitrocellulose membrane (LifeScience). The membrane was blocked with 5% BSA in TBS-T for 1h at room temperature, and in order to evaluate a possible processing at the protein level, it was incubated with FLAG antibody (1:20000) 1h at room temperature. After incubation with Horseradish peroxidase conjugated anti-Mouse IgG, membrane was developed using the EZ-ECL chemiluminescence detection kit. Naphtol blue staining was used to verify equal loading.
(TIF)
