# Supplementary figures and images for: Identification and characterization of new isoforms of human fas apoptotic inhibitory molecule (FAIM)
Source: PLoS One. 2017 Oct 5;12(10):e0185327. doi: 10.1371/journal.pone.0185327 (PMC5628826; doi:10.1371/journal.pone.0185327)

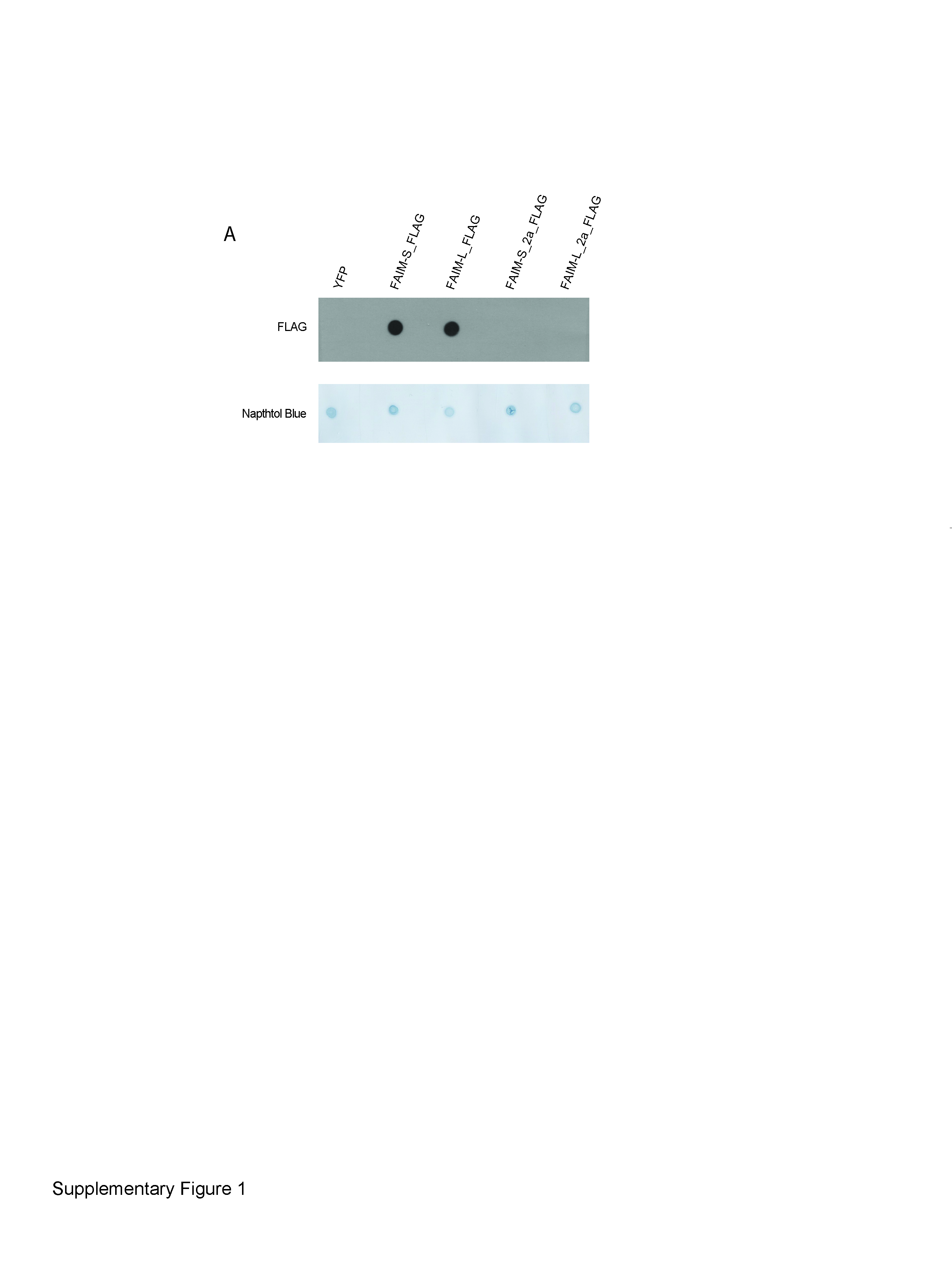

Supplement: S1 Fig — HEK293T cells were transiently transfected with the four FLAG-tagged isoforms (Fig 5C). 2.5μg of lysate was spotted on a Nitrocellulose membrane (LifeScience). The membrane was blocked with 5% BSA in TBS-T for 1h at room temperature, and in order to evaluate a possible processing at the protein level, it was incubated with FLAG antibody (1:20000) 1h at room temperature. After incubation with Horseradish peroxidase conjugated anti-Mouse IgG, membrane was developed using the EZ-ECL chemiluminescence detection kit. Naphtol blue staining was used to verify equal loading. (TIF) [file pone.0185327.s001.tif]
